# Generalized higher (*U*,*M*)-derivations in prime Γ-Rings

**DOI:** 10.1186/2193-1801-3-100

**Published:** 2014-02-19

**Authors:** Md Mizanor Rahman, Akhil Chandra Paul

**Affiliations:** Department of Mathematics, Jagannath University, Dhaka, Bangladesh; Department of Mathematics, University of Rajshahi, Rajshahi, Bangladesh

**Keywords:** Lie ideal, Admissible Lie ideal, (*U, M*)-derivation, Generalized (*U, M*)-derivation, Generalized higher (*U, M*)-derivation, Prime Γ-ring

## Abstract

Let *M* be a 2‐torsion free prime Γ‐ring satisfying the condition *aαbβc*=*aβbαc*,∀*a*,*b*,*c*∈*M* and *α*,*β*∈Γ, *U* be an admissible Lie ideal of *M* and *F*=(*f*_*i*_)_*i*∈**N**_ be a generalized higher (*U*,*M*)‐derivation of *M* with an associated higher (*U*,*M*)‐derivation *D*=(*d*_*i*_)_*i*∈**N**_ of *M*. Then for all *n*∈**N** we prove that .

**Mathematics Subject Classification (2010)**: 13N15; 16W10; 17C50

## Introduction

The notion of a Γ‐ring has been developed by Nobusawa (1964), as a generalization of a ring. Following Barnes (1966) generalized the concept of Nobusawa’s Γ‐ring as a more general nature. Nowadays Γ‐ring theory is a showpiece of mathematical unification, bringing together several branches of the subject. It is the best research area for the Mathematicians and during 40 years, many classical ring theories have been generalized in Γ‐rings by many authors. The notions of derivation and Jordan derivation in Γ‐rings have been introduced by Sapanci and Nakajima (1997). Afterwards, in the light of some significant results due to Jordan left derivation of a classical ring obtained by Jun and Kim (1996), some extensive results of left derivation and Jordan left derivation of a Γ‐ring were determined by Ceven (2002). In (Halder and Paul 2012), Halder and Paul extended the results of (Ceven 2002) in Lie ideals. Let *M* and Γ be additive abelian groups. If there is a mapping *M*×Γ×*M*→*M* (sending (*x*,*α*,*y*) into *xαy*) such that (i) (*x*+*y*)*αz*=*xαz*+*yαz*,*x*(*α*+*β*)*y*=*xαy*+*xβy*,*xα*(*y*+*z*)=*xαy*+*xαz*, (ii) (*xαy*)*βz*=*xα*(*yβz*), for all *x*,*y*,*z*∈*M* and *α*,*β*∈Γ, then *M* is called a Γ‐*ring*. This concept is more general than a ring and was introduced by Barnes (1966). A Γ‐ring *M* is called a *prime Γ‐ring* if ∀*a*,*b*∈*M*,*a*Γ*M*Γ*b*=0 implies *a*=0 or *b*=0. A Γ‐ring *M* is *2‐torsion free* if 2*a*=0 implies *a*=0,∀*a*∈*M*. For any *x*,*y*∈*M* and *α*∈Γ, we induce a new product, the *Lie product* by [ *x*,*y*]_*α*_=*xαy*−*yαx*. An additive subgroup *U*⊂*M* is said to be a *Lie ideal* of *M* if whenever *u*∈*U*,*m*∈*M* and *α*∈Γ, then [ *u*,*m*]_*α*_∈*U*. In the main results of this article we assume that the Lie ideal *U* verifies *uαu*∈*U*,∀*u*∈*U*. A Lie ideal of this type is called a *square closed Lie ideal*. Furthermore, if the Lie ideal *U* is square closed and *U* is not contained in *Z*(*M*), where *Z*(*M*) denotes the center of *M*, then *U* is called an *admissible Lie ideal* of *M*. In (Herstein 1957), Herstein proved a well‐known result in prime rings that every Jordan derivation is a derivation. Afterwards many Mathematicians studied extensively the derivations in prime rings. In (Awter 1984), Awtar extended this result in Lie ideals. (*U*,*R*)‐derivations in rings have been introduced by Faraj et al. (2010), as a generalization of Jordan derivations on a Lie ideals of a ring. The notion of a (*U*,*R*)‐derivation extends the concept given in (Awter 1984). In this paper (Faraj et al. 2010), they proved that if *R* is a prime ring, char (*R*)≠2, *U* a square closed Lie ideal of R and d a (*U*,*R*)‐ derivation of *R*, then *d*(*ur*)=*d*(*u*)*r*+*ud*(*r*),∀,*u*∈*U*,*r*∈*R*. This result is a generalization of a result in (Awter (1984), Theorem in section 3). In this article, we introduce the concept of a (*U*,*M*)‐derivation, generalized (*U*,*M*)‐derivation and generalized higher (*U*,*M*)‐derivation, where *U* is a Lie ideal of a Γ‐ring *M*. Examples of a Lie ideal of a Γ‐ring, (*U*,*M*)‐derivation, generalized (*U*,*M*)‐derivation, higher (*U*,*M*)‐derivation and generalized higher (*U*,*M*)‐derivation are given here. A result in (Halder and Paul (2012), Theorem 2.8) is generalized in Γ‐rings by the new concept of a (*U*,*M*)‐derivation. Throughout the article, we use the condition *aαbβc*=*aβbαc*,∀*a*,*b*,*c*∈*M* and *α*,*β*∈Γ and this is represented by (*). We make the basic commutator identities [ *xαy*,*z*]_*β*_= [ *x*,*z*]_*β*_*αy*+*x*[ *α*,*β*]_*z*_*y*+*xα*[*y*,*z*]_*β*_, [ *x*,*yαz*]_*β*_= [ *x*,*y*]_*β*_*αz*+*y*[ *α*,*β*]_*x*_*z*+*yα*[ *x*,*z*]_*β*_, ∀*x*,*y*,*z*∈*M*,∀*α*,*β*∈Γ. According to the condition (*), the above two identities reduces to [ *xαy*,*z*]_*β*_= [ *x*,*z*]_*β*_*αy*+*xα*[*y*,*z*]_*β*_,[ *x*,*yαz*]_*β*_= [ *x*,*y*]_*β*_*αz*+*yα*[ *x*,*z*]_*β*_,∀*x*,*y*,*z*∈*M*,∀*α*,*β*∈Γ.

## Generalized (*U*,*M*)‐derivation

In view of the concept of (*U*,*R*)‐derivation of an ordinary ring developed by Faraj et al. (2010), we have been determined some important results in Rahman and Paul (2013) due to these concepts in case of certain Γ‐rings after introducing the notions of (*U*,*M*)‐derivation of Γ‐rings as defined below.

### Definition 1

(Rahman and Paul (2013), Definition 2.1) Let *M* be a Γ‐ring and *U* be a Lie ideal of *M*. An additive mapping *d* :*M*→*M* is said to be a *(U*,*M*)‐*derivation* of *M* if *d*(*uαm* + *sαu*) = *d*(*u*)*αm* + *uαd*(*m*) + *d*(*s*)*αu* + *sαd*(*u*),∀*u*∈*U*,*m*,*s*∈*M* and *α*∈Γ.

### Definition 2.

(Rahman and Paul (2013), Definition 2.2) Let *M* be a Γ‐ring and *U* be a Lie ideal of *M*. An additive mapping *f* :*M*→*M* is said to be a *generalized (U, M)‐ derivation* of *M* if there exists a (*U*,*M*)‐derivation *d* of *M* such that *f*(*uαm*+*sαu*)=*f*(*u*)*αm*+*uαd*(*m*)+*f*(*s*)*αu*+*sαd*(*u*),∀*u*∈*U*,*m*,*s*∈*M* and *α*∈Γ.

The existence of a Lie ideal of a Γ‐ring, (*U*,*M*)‐derivation and a generalized (*U*,*M*)‐derivation are confirmed by the following examples.

### Example 1.

Let *R* be an associative ring with 1 and *U* a Lie ideal of *R*. Let *M*=*M*_1,2_(*R*) and , then *M* is a Γ‐ring.

If *N*={(*x*,*x*):*x*∈*R*}⊆*M* and *U*_1_={(*u*,*u*):*u*∈*U*} then *N* is a sub Γ‐ring of *M* and *U*_1_ is a Lie ideal of *N*. Let *f*:*R*→*R* be a generalized (*U*,*R*)‐derivation. Then there exists a (*U*,*R*)‐derivation *d*:*R*→*R* such that *f*(*uαx*+*sαu*)=*f*(*u*)*αx*+*uαd*(*x*)+*f*(*s*)*αu*+*sαd*(*u*).

If we define a mapping *D* :*N*→*N* by *D*((*x*,*x*))=(*d*(*x*),*d*(*x*)), then we have .

After calculation we have *D*(*u*_1_*αx*_1_+*y*_1_*αu*_1_)=*D*(*u*_1_)*αx*_1_+*u*_1_*αD*(*x*_1_)+*D*(*y*_1_)*αu*_1_+*y*_1_*αD*(*u*_1_), where .

Hence *D* is a (*U*_1_,*N*)− derivation on *N*.

Let *F* :*N*→*N* be the additive mapping defined by *F*((*x*,*x*))=(*f*(*x*),*f*(*x*)), then considering , we have .

Hence *F* is a generalized (*U*_1_,*N*)−derivation on *N*.

### Lemma 1

(Rahman and Paul (2013), Lemma 2.4) Let M be a 2‐torsion free Γ‐ring satisfying the condition (*). U be a Lie ideal of M and f be a generalized (U,M)‐derivation of M. Then

(i) *f(uαmβu)=f(u)αmβu*+*uαd*(*m*)*βu*+*uαmβd*(*u*),∀*u*∈*U*,*m*∈*M and α*,*β*∈Γ.
(ii) *f*(*uαmβv*+*vαmβu*)=*f*(*u*)*αmβv+uαd*(*m*)*βv*+*uαmβd*(*v*)+*f*(*v*)*αmβu*+*vαd*(*m*)*βu*+*vαmβd*(*u*),∀*u*,*v*∈*U*,*m*∈*M and α*,*β*∈Γ.

### Definition 3.

(Rahman and Paul (2013), Definition 2.5) Let *d* be a (*U*,*M*)‐derivation of *M*, then we define Φ_*α*_(*u*,*m*)=*d*(*uαm*)−*d*(*u*)*αm*−*uαd*(*m*),∀*u*∈*U*,*m*∈*M* and *α*∈Γ.

Now, we state some useful results that have already been discussed in Rahman and Paul (2013).

### Lemma 2.

*Let d be a* (*U*,*M*)‐*derivation of M, then*

(i) Φ_*α*_ (*u*,*m*)=−Φ_*α*_(*m*,*u*), ∀*u*∈*U*,*m*∈*M and α*∈Γ.
(ii) Φ_*α*_(*u*+*v*,*m*)=Φ_*α*_(*u*,*m*)+Φ_*α*_(*v*,*m*),∀*u*,*v*∈*U*,*m*∈*Mand α*∈Γ.
(iii) Φ_*α*_(*u*,*m*+*n*)=Φ_*α*_(*u*,*m*)+Φ_*α*_(*u*,*n*),∀*u*∈*U*,*m*,*n*∈*M and α*∈Γ.
(iv) Φ_*α*+*β*_(*u*,*m*)=Φ_*α*_(*u*,*m*)+Φ_*β*_(*u*,*m*),∀*u*∈*U*,*m*∈*M and α*,*β*∈Γ.

The proofs are obvious by using the Definition 3.

### Definition 4.

(Rahman and Paul (2013), Definition 2.7) If *f* is a generalized (*U*,*M*)‐derivation of *M* and *d* is a (*U*,*M*)‐derivation of *M*, then we define *Ψ*_*α*_(*u*,*m*)=*f*(*uαm*)−*f*(*u*)*αm*−*uαd*(*m*),∀*u*∈*U*,*m*∈*M* and *α*∈Γ.

Also, we need the following important results that have already been discussed in Rahman and Paul (2013).

### Lemma 3.

*Let f be a generalized* (*U*,*M*)‐*derivation of M, then*

(i) *Ψ*_*α*_(*u*,*m*)=−*Ψ*_*α*_(*m*,*u*),∀*u*∈*U*,*m*∈*Mand α*∈Γ.
(ii) *Ψ*_*α*_(*u*+*v*,*m*)=*Ψ*_*α*_(*u*,*m*)+*Ψ*_*α*_(*v*,*m*),∀*u*,*v*∈*U*,*m*∈*Mand α*∈Γ.
(iii) *Ψ*_*α*_(*u*,*m*+*n*)=*Ψ*_*α*_(*u*,*m*)+*Ψ*_*α*_(*u*,*n*),∀*u*∈*U*,*m*,*n*∈*M and α*∈Γ.
(iv) *Ψ*_*α*+*β*_(*u*,*m*)=*Ψ*_*α*_(*u*,*m*)+*Ψ*_*β*_(*u*,*m*),∀*u*∈*U*,*m*∈*M and α*,*β*∈Γ.

The proofs are obvious by using the Definition 4.

### Lemma 4.

(Rahman and Paul (2013), Lemma 2.11) Let U be a Lie ideal of a 2‐torsion free prime Γ‐ring M satisfying the condition (*) and U is not contained in Z(*M*). *If a,b∈M (resp. b*∈*U and a∈M) such that a αUβb=0,∀α*,*β*∈Γ, *then a*=0 *or b*=0.

### Theorem 1.

(Rahman and Paul (2013), Theorem 2.13) Let M be a 2‐torsion free prime Γ‐ring satisfying the condition (*), U be an admissible Lie ideal of M and f be a generalized (U,M)‐derivation of M, then Ψ_*α*_(*u*,*v*)=0,∀*u*,*v*∈*U and α*∈Γ.

### Remark 1

If we replace *U* by a square closed Lie ideal in the Theorem 1, then the theorem is also true.

### Theorem 2.

(Rahman and Paul (2013), Theorem 2.14) Let M be a 2‐torsion free prime Γ‐ring satisfying the condition (*), U a square closed Lie ideal of M and f be a generalized (U,*M*)‐*derivation of M, then f*(*uαm*)=*f*(*u*)*αm*+*uαd*(*m*),∀*u*∈*Um*∈*M and α*∈Γ.

## Generalized higher (*U*,*M*)‐derivation

In this section, we introduce generalized higher (*U*,*M*)‐derivations in Γ‐rings.

### Definition 5.

Let *M* be a Γ‐ring and *U* be a Lie ideal of *M* and  be a family of additive mappings of *M* into itself such that *f*_0_=*id*_*M*_, where *id*_*M*_ is an identity mapping on *M*. Then *F* is said to be a *generalized higher (U*,*M)‐derivation* of *M* if there exists an higher (*U*,*M*)‐derivation *D*=(*d*_*i*_)_*i*∈**N**_ of *M* such that for each  and *α*,*β*∈Γ.

### Example 2.

Let *N* and *U*_1_ are as in Example 1. If *f*_*n*_ :*R*→*R* be a generalized higher (*U*,*R*)‐derivation. Then there exists a higher (*U*_1_,*R*) derivation *d*_*n*_ :*R*→*R* such that .

If we define a mapping *D*_*n*_ :*N*→*N* by *D*_*n*_((*x*,*x*))=(*d*_*n*_(*x*),*d*_*n*_(*x*)). Then *D*_*n*_ is a higher (*U*_1_,*N*)‐derivation on *N*.

Let *F*_*n*_ :*N*→*N* be the additive mapping defined by *F*_*n*_((*x*,*x*))=(*f*_*n*_(*x*),*f*_*n*_(*x*)). Then by the similar calculation as in Example 1, we can show that, *F*_*n*_ is a generalized higher (*U*_1_,*N*)‐derivation on *N*.

### Lemma 5.

Let M be a 2‐torsion free Γ‐ring satisfying the condition (*), U be a Lie ideal of M and F=(*f*_*i*_)_*i*∈***N***_*be a generalized higher (U,M)‐derivation of M. Then**and α,β*∈Γ.

### Proof

Let *x*=*uα*((2*u*)*βm*+*mβ*(2*u*))+((2*u*)*βm*+*mβ*(2*u*))*αu*.

Replacing *m* and *s* by (2*u*)*βm*+*mβ*(2*u*) and (2*u*)*αm*+*mα*(2*u*) respectively in  and using the condition (*), we have .

Thus we have
1

On the other hand by the definition of higher (*U*,*M*)‐ derivation and using the condition (*) .

Thus we have
2

Now comparing (1) and (2) we get  and *α*,*β*∈Γ. Using 2‐torsion freeness of *M*, we get the desired result.

### Lemma 6.

*Let M be a 2‐torsion free* Γ‐ring satisfying the condition (*), U be a Lie ideal of M and F=(*f*_*i*_)_*i*∈***N***_*be a generalized higher (U,M)‐derivation of M. Then**and α,β*∈Γ.

### *Proof*.

Linearizing of  with respect to *u* gives us .

On the other hand .

Now comparing above two expressions, we get  and *α*,*β*∈Γ.

### Definition 6.

Let *M* be a 2‐torsion free Γ‐ring satisfying the condition (*) and *U* be a Lie ideal of *M*. Let *F*=(*f*_*i*_)_*i*∈**N**_ be a generalized higher (*U*,*M*)‐derivation of *M*. For every fixed *n*∈**N**, we define . Also let *D*=(*d*_*i*_)_*i*∈**N**_ be a higher (*U*,*M*)‐derivation of *M*. For every fixed *n*∈**N**, we define .

### Remark 2.

 and *n*∈**N** if and only if  and *n*∈**N**. Also  and *n*∈**N** if and only if  and *n*∈**N**.

### Lemma 7.

Let M be a 2‐torsion free Γ‐ring satisfying the condition (*) and U be a Lie ideal of M. For every u∈U,m∈M,α∈Γ and n∈***N***, *then**and*.

The proofs are obvious by the Definition 6, higher (*U*,*M*)‐derivation of *M* and generalized higher (*U*,*M*)‐derivation of *M*.

### Lemma 8.

Let M be a 2‐torsion free prime Γ‐ring satisfying the condition (*), U be an admissible Lie ideal of M and F=(*f*_*i*_)_*i*∈***N***_*be a generalized higher (U,M)‐derivation of M. Then**and n*∈***N***.

### *Proof*.

We have  and by Theorem 1, .

Now we assume, by induction on *n*∈**N**, that , *m*∈**N** and *m*<*n*.

Let *x*=4(*uαvβwγvαu*+*vαuβwγuαv*).

Then by using Lemma 6, we have .

On the other hand, by Lemma 5 and *D*=(*d*_*i*_)_*i*∈**N**_ is a higher (*U*,*M*)‐derivation of *M*. .

Now comparing the two expressions of *f*_*n*_(*x*) and using , we get .

Using Lemma 7 and 2‐torsion freeness of *M* we get .

Since *D*=(*d*_*i*_)_*i*∈**N**_ is a higher (*U*,*M*)‐derivation of *M*, thus we have . Now by Lemma 4 and since *U* is noncentral, thus we get  and *n*∈**N**.

Now we prove the main result.

### Theorem 3.

Let M be a 2‐torsion free prime Γ‐ring satisfying the condition (*), U be an admissible Lie ideal of M and F=(*f*_*i*_)_*i*∈**N**_*be a generalized higher (U,M)‐derivation of M. Then**and n*∈***N***.

### *Proof*.

We have  and by Theorem 1, .

Now we assume, by induction on *n*∈**N**, that  and *m*<*n*.

Now since *F*=(*f*_*i*_)_*i*∈**N**_ is a generalized higher (*U*,*M*)‐derivation of *M*, we have .

Since *D*=(*d*_*i*_)_*i*∈**N**_ is a higher (*U*,*M*)‐derivation of *M*, thus we have
3

Since *F*=(*f*_*i*_)_*i*∈**N**_ is a generalized higher (*U*,*M*)‐derivation of *M*, thus we have .
4

Since .
5

On the other hand, by using Equation (3) and Lemma 5, we get .
6

By comparing (5) and (6) and using the condition (*), we get
7

Linearizing of (7) with respect to *u*, gives us
8

Replacing *v* by *vαv* in (8) and since , thus . This implies that .

Hence by Lemma 4 and since  and *n*∈**N**.

Thus by the Remark 2, we have  and *n*∈**N**.
